# Predictive Molecular Biomarkers of Bladder Cancer Identified by Next-Generation Sequencing—Preliminary Data

**DOI:** 10.3390/jcm13247701

**Published:** 2024-12-17

**Authors:** Aleksander Myszka, Marek Ciesla, Aleksandra Siekierzynska, Anna Sendera, Constantina Constantinou, Pawel Karpinski, Grzegorz Wysiadecki, Krzysztof Balawender

**Affiliations:** 1 Institute of Medical Sciences, University of Rzeszow, 35-310 Rzeszow, Poland; mciesla@ur.edu.pl (M.C.); atrzyna@ur.edu.pl (A.S.); kbalawender@ur.edu.pl (K.B.); 2 Department of Biotechnology and Plant Physiology, University of Rzeszow, 35-601 Rzeszow, Poland; asiekierzynska@ur.edu.pl; 3 Medical School, University of Nicosia, Nicosia 2414, Cyprus; constantinou.co@unic.ac.cy; 4 Department of Genetics, Wroclaw Medical University, 50-368 Wroclaw, Poland; pawel.karpinski@umed.wroc.edu.pl; 5 Department of Normal and Clinical Anatomy, Medical University of Lodz, 90-752 Łodz, Poland; grzegorz.wysiadecki@umed.lodz.pl

**Keywords:** molecular biomarkers, bladder cancer recurrence, somatic mutations, disease-free survival

## Abstract

**Background:** The majority of patients with bladder cancer suffer from tumour recurrence. Identifying prognostic factors for tumour recurrence is crucial for treatment and follow-up in affected patients. The study aimed to assess the impact of somatic mutations in bladder cancer on patient outcomes and tumour recurrence. **Methods:** The study group comprised 46 patients with urothelial bladder cancers referred for transurethral resection of the tumour. A molecular study on tumour-derived DNA was performed using next-generation sequencing. Somatic mutations were screened in 50 genes involved in carcinogenesis. **Results:** We identified 81 variants in 23 genes, including 54 pathogenic mutations, 18 likely pathogenic variants, and 9 variants of unknown significance. The most frequently mutated genes were *FGFR3*, *PIK3CA*, and *TP53* in 52%, 35%, and 24% of tumours, respectively. The average tumour-free survival was significantly longer in cases with mutations in the *PIK3CA* gene (*p* = 0.02), and mutations in the *PIK3CA* gene were associated with a decreased risk of tumour recurrence (Hazard Ratio = 0.26; 95% CI: 0.11–0.62; *p* = 0.018). **Conclusions:** The *PIK3CA* gene was shown to be a predictive marker of a low risk of bladder tumour recurrence. Molecular screening of bladder cancers supported predictive biomarkers of tumour recurrence and showed that tumour-free survival is molecularly determined.

## 1. Introduction

Bladder cancer is the 10th most common cancer diagnosed worldwide. The incidence of bladder cancer is four times higher in men than in women, and the cumulative risk is 1.05% and 0.26% in men and women, respectively. Mortality caused by bladder cancer is 0.18% in men and 0.8% in women [1]. While the 5-year relative survival rate for bladder cancer is relatively high at 77% across all stages [2], tumour recurrence is high, affecting 30–80% of patients [3]. Therefore, looking for predictive biomarkers for bladder cancer recurrence is necessary.

In bladder tumorigenesis, two main molecular pathways are involved. In low-grade papillary carcinoma, mutations in the *FGFR3* or *RAS* genes are involved, while in high-grade in situ/muscle-invasive carcinoma, mutations are present in the *TP53* and *RB1* genes. Mutations in the mentioned genes lead to the accumulation of mutations in other genes and aggressive cancer [4]. Some genomic variants are known targets for bladder cancer drugs, e.g., the phosphatidylinositol-3-OH kinase/AKT/mTOR pathway, the RTK/MAPK pathway, and ERBB2 [5].

Nowadays, the identification of molecular biomarkers is facilitated by high-throughput sequencing techniques. These techniques enable the comprehensive characterisation of germline and somatic mutations across numerous genes during one experiment. Identifying specific variants and disturbances of the cellular pathways may indicate predictive biomarkers and targeted therapies. Despite the potential benefits, only a limited number of studies in bladder cancer have been performed using massively parallel sequencing technology known as next-generation sequencing (NGS). Given the significant risk of bladder cancer recurrence, establishing predictive molecular biomarkers is of paramount importance.

Our study aimed to assess the impact of somatic mutations in bladder cancer on patient outcomes and tumour recurrence.

## 2. Materials and Methods

### 2.1. Study Participants

The patients who participated in the study were diagnosed with urothelial carcinoma. Patients were consecutively recruited before TURBT treatment (Transurethral Resection of Bladder Tumour) at the Provincial Hospital in Zamosc, Poland. The cohort consisted of 46 subsequent patients with urothelial carcinoma, 36 patients affected with non-muscle-invasive bladder cancer (NMIBC), and 10 with muscle-invasive bladder cancer (MIBC). Eight patients underwent treatment with BCG (Bacillus Calmette-Guerin). The clinical characteristics of the patients are presented in Table 1. All participants gave their informed consent to participate in this research programme, which was approved by the University of Rzeszow Ethics Committee (no. 2022/018).

### 2.2. TURBT and Tumour Extraction

The tumours were extracted using TURBT, a minimally invasive procedure performed through a cystoscope. The extracted tumours were formalin-fixed and embedded in paraffin. A pathologist confirmed the diagnosis of bladder cancer and assessed tumour staging and grading based on haematoxylin and eosin-stained slides.

### 2.3. Mutation Screening

Somatic mutation screening was performed using tumour-derived DNA. DNA was purified using a Sherlock AX Kit (A&A Biotechnology, Gdansk, Poland) according to the manufacturer’s instructions and quantified with the Qubit 4 instrument using the Qubit dsDNA Quantitation High Sensitivity Kit (both from Thermo Fisher Scientific, Waltham, MA, USA). Then, 10 ng of total DNA was used for the construction of the targeted library and prepared using the AmpliSeqTMCancer Hotspot Panel v2 and AmpliSeqTMLibrary Kit 2.0 (Thermo Fisher Scientific, Waltham, CA, USA), according to the manufacturer’s protocol. The pool of 207-pair primers covering approximately 2800 COSMIC mutations from 50 oncogenes and tumour suppressor genes was amplified. Libraries were evaluated on the Agilent 2100 Bioanalyser (Agilent Technologies, Inc., Santa Clara, CA, USA) using the High Sensitivity DNA Kit (Agilent Technologies, Inc., Santa Clara, CA, USA). Sequencing was performed on the Ion S5 System (Thermo Fisher Scientific, Waltham, CA, USA) using Ion Torrent superconductor technology. The Ion Reporter™ software (version 5.16) was used to annotate variants. Identified variants matched the following criteria: allele frequency in tissue from 0.05 to 1 (5–100%), variants not included in the UCSC Genome Browser Common SNPs database, and minor allele frequencies <0.01 in the dbSNP database of the 1000 Genomes Project. The clinical significance of the variants and their pathogenicity was determined based on their classification in the ClinVar database [6] and in the VarSome database based on the ACMG classification (version: 11.2.7) [7].

### 2.4. Statistical Analysis

One-way analysis of variance (ANOVA) was used to assess differences between groups. Pearson’s and Spearman’s coefficients were used to evaluate correlations between variables. The LogRank test was used to compare survival curves. Univariate Cox regression analysis was used to calculate the hazard ratio (HR). Hierarchical Cluster Analysis was performed using the Ward method. *p*-values < 0.05 were considered statistically significant. Statistical analysis was performed using PQStat Software v.1.8.6. (Poznan, Poland).

## 3. Results

**Figure 1:**
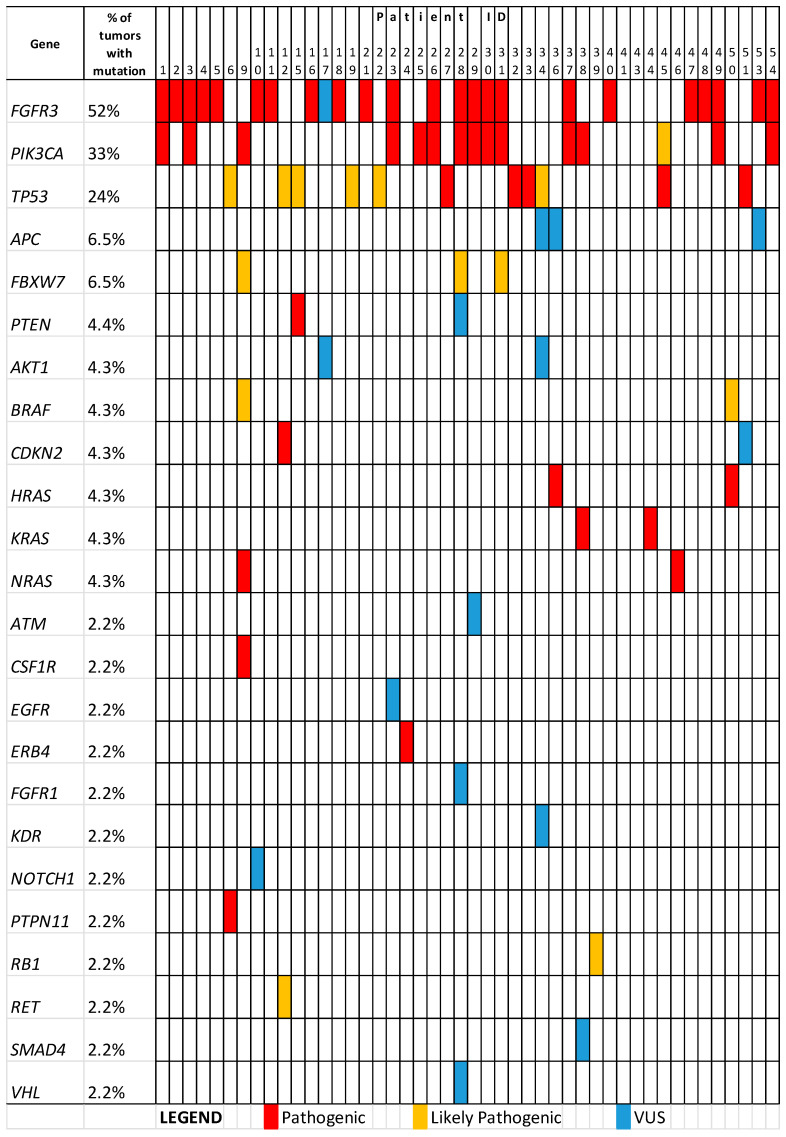
The prevalence of variants identified in bladder tumours of studied patients.

Targeted sequencing identified 54 pathogenic variants, 18 likely pathogenic variants, and 8 rare missense substitutions currently classified as VUS (variants of unknown significance). The verification of variant classification using public databases is presented in Table S1 in the Supplementary Materials. A total of 81 variants were revealed in 23 genes in 44 tumour samples (Figure 1). Samples from two patients did not have any variant in the analysed genes. Therefore, pathogenic/likely pathogenic variants or VUS were observed in 95% of tumours.

The most frequently mutated genes were *FGFR3*, *PIK3CA*, and *TP53* in 52%, 33%, and 24% of tumours, respectively (Figure 1). The spectrum of detected variants is listed in Table 2.

**Figure 2:**
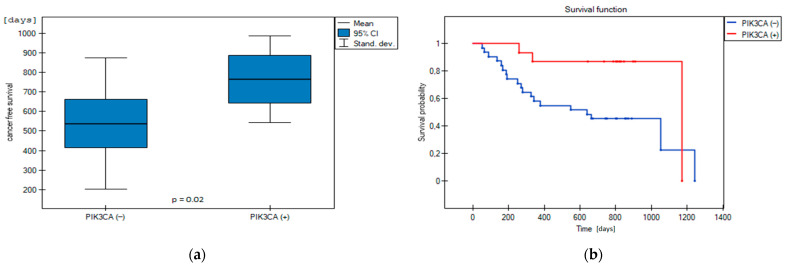
Comparison of cancer-free survival between patients with somatic mutations (+) and patients without somatic mutations (−) in the *PIK3CA* gene: (**a**) recurrence-free survival in patients with mutations compared to patients without mutation; (**b**) impact of mutations in the *PIK3CA* gene on cancer-free survival probability of bladder cancer patients.

We demonstrated that the average recurrence-free survival was significantly longer in patients with mutations in the *PIK3CA* gene (*n* = 16.35%) compared to patients without mutation (*n* = 30.65%) (average 764 vs. 537 days, respectively, *p* = 0.02, Figure 2a). Furthermore, mutations in the *PIK3CA* gene were associated with a decreased risk of tumour recurrence (Hazard Ratio: 0.26; 95% CI: 0.11–0.62; *p* = 0.018). Cancer-free survival curves differ significantly between mutation patients compared to mutation-free cases in this gene (Figure 2b).

**Figure 3:**
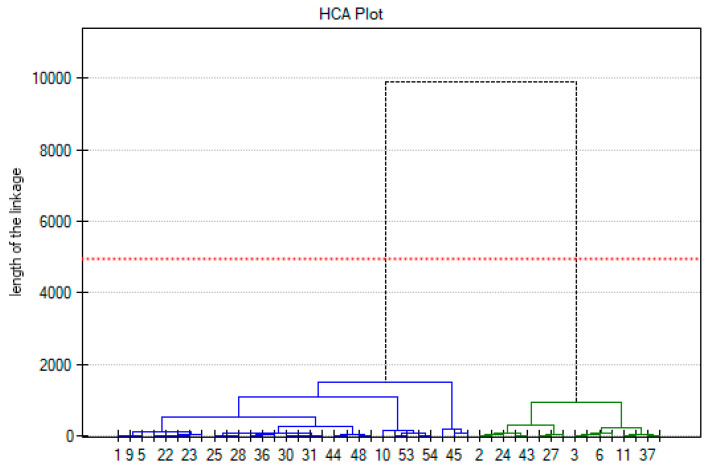
Hierarchical Cluster Analysis covering age of onset, cancer-free survival and a number of pathogenic mutations. Clusters differ significantly with respect to survival (*p* < 0.001). Blue and green lines indicate extracted clusters. The red dotted line indicates the cutoff level set at 50% of the length of the longest bond in the dendrogram and determines the number of clusters.

Hierarchical Cluster Analysis covering the age of onset, cancer-free survival, and the number of pathogenic mutations revealed two main clusters (Figure 3) that differed significantly according to survival rate (*p* < 0.001).

The study’s results prove an inverse correlation between age of onset and cancer-free survival (Spearman coefficient r = −0.3 5; *p* = 0,01).

In patients with a mutation in the *TP53* gene, we observed a higher tumour grade than patients without mutations in this gene (*p* = 0.02).

We also observed an inverse correlation between the age of onset and recurrence-free survival (r = −0.3; *p* = 0.01).

## 4. Discussion

Tumour recurrence, in addition to tumour size, stage, grade, focality, and presence of CIS, is one of the clinical determinants of cancer progression [3]. In patients with bladder cancer, tumour recurrence is very common, and it is estimated that up to 80% of patients have a high risk of cancer recurrence [3,8,9]. In our cross-sectional study, tumour recurrence occurred in 48% of the cases, confirming the need to search for cancer progression biomarkers. NGS could allow the identification of targeted and prognostic molecular markers of bladder cancer [9].

Our research identified pathogenic/likely pathogenic variants or VUS in 95% of tumours. Mutations in the *FGFR3* gene were detected in 52% of tumours, mutations in *PIK3CA* in 35% of tumours, and mutations in *TP53* in 24% of tumours. Mutations in other genes occurred rarely. In other research, these genes were also frequently mutated in urothelial cancers; however, gene spectrum and mutation incidence differ between studies (Table 3). The differences probably result from selection criteria of the type of bladder cancer, study sample sizes, and number of studied genes (Table 3).

We recognised hotspot variants in two genes, *FGFR3* (c.746C>G; p.Ser249Cys) and *PIK3CA* (c.1633G>A; p.Glu545Lys). This observation is consistent with other studies [15]. In the *TP53* gene, we did not observe hotspot variants. This gene is the most frequently mutated in metastatic urothelial carcinoma (57% of patients), with E285K being the highest-frequency hotspot variant [15]. Similarly, mutations in *RB1* in our study were sporadic, but in patients with metastatic urothelial carcinoma, mutations in the *RB1* gene were more frequent (18%) [15].

Our study confirmed the presence of molecular clinical markers of bladder cancer. We found that mutations in the *TP53* gene were associated with a higher tumour grade (*p* = 0.02). This observation complies with a previous study by Pietzak et al. [10]. In our study, we note that the presence of mutations in *TP53* has ruled out the co-occurrence of mutations in *FGFR3* (see Figure 1), which confirms the activation of different pathways of carcinogenesis in tumours.

In this study, we showed that *PIK3CA* mutations are associated with a reduced risk of bladder cancer recurrence in patients from the Central European population. So far, this association has been found in the Spanish population (87 cases) [16] and the USA population (109 cases) [17]. Since the association of *PIK3CA* mutations with disease recurrence could have clinical implications, confirmation of the observed association in large cohorts from different populations is needed.

We demonstrated that the average recurrence-free survival was significantly longer in patients with mutations in the *PIK3CA* gene compared to patients without mutations in this gene (764 vs. 537 days, respectively, *p* = 0.02, Figure 2). Furthermore, mutations in the *PIK3CA* gene were associated with a lower risk of tumour recurrence (Hazard Ratio HR = 0.26; 95% CI: 0.11–0.62; *p* = 0.018). Cancer-free survival curves differ between patients with mutation compared to cases without mutation in this gene (Figure 3). Patients with variants in the *PIK3CA* gene have a better probability of recurrence-free survival. The protective effect of *PIK3CA* mutations is related to a reduced probability of disease recurrence by about 74% in the studied group. Concerning the entire population of patients with bladder cancer, the effect of the *PIK3CA* mutation with a 95% probability will be beneficial, with the HR range of 0.11–0.62. Reducing the probability of disease recurrence by 38–89% is an argument for implementing the *PIK3CA* gene as a predictive biomarker of disease recurrence in clinical practice and further validation. A similar observation was reported by Dueñas et al. [16] in patients with bladder cancer. They reported that alterations in the *PIK3CA* gene (mutations and copy gains) were associated with a reduced probability of tumour recurrence. An analogous observation was made by Kim et al. [17], which provided evidence that *PIK3CA* mutations were associated with a higher recurrence-free survival (HR: 0.35; *p* = 0.01) and better cancer-specific survival (HR: 0.35; *p* = 0.04) in patients treated with radical cystectomy. Furthermore, *PIK3CA* mutations in multivariable analyses controlling for pT and pN stages were also associated with better recurrence-free survival (HR: 0.39; *p* = 0.03) [17].

Although mutations in the PIK3CA gene were correlated with a lower risk of relapse, the role of mutations in this gene has not been fully explained. It has been noted that *FGFR3* mutations often accompany mutations in the *PIK3A* gene. Patients with *FGRF3* mutated and *PIK3CA* wild type showed worse recurrence rates compared to *FGFRG3* mutated and *PIK3CA* mutated genotype [16]. This co-occurrence suggests a complex interaction between these genes, but it does not establish *PIK3CA* as a standalone predictive marker for recurrence-free survival in bladder cancer patients.

In some studies, mutations in the *PIK3CA* gene were not associated with recurrence-free survival. The study conducted by Kompier et al. [18] showed that mutations in *RAS* and *PIK3CA* genes were not markers for recurrence-free survival, progression-free survival, or disease-specific survival in bladder cancer patients. Also, the study by Ward et al. [12] did not demonstrate that *PIK3CA* mutations are prognostic. Another study conducted in patients from the Moroccan population showed no correlation between the mutation status in the *PIK3CA* gene and tumour recurrence [19]. Notably, the authors indicated that a limitation of the study was the low frequency of mutations in the *PIK3CA* gene (approximately 13%), which does not reflect the general occurrence of these variants in bladder cancer cases. This observation may suggest that the mutation incidence and clinical significance may relate to the studied population. Another study conducted by Critelli et al. [20] also did not identify the *PIK3CA* gene as a potential marker associated with the prediction of recurrence. They indicated that other genes, *TERT* and *FGFR3*, were most closely associated with tumour recurrence [20]. It seems further studies are needed on the *PIK3CA* gene to determine its significance in bladder cancer.

Somatic mutations’ impact on survival and recurrence may vary across populations or ethnic groups. There is growing evidence that pathogenic variants do not have uniform prognostic implications across all patient groups. Germline polymorphisms can significantly shape tumour characteristics. Such evidence was provided by research *TP53* mutations and polymorphisms in breast cancer, conducted by Hebert-Magee et al. [21]. This study highlighted that the survival impact of *TP53* somatic mutations is influenced by ethnicity and molecular subtype. African American women with the Pro/Pro codon 72 variant and a *TP53* missense mutation had significantly worse survival outcomes, particularly in luminal subtypes. This interaction between somatic mutations and population-specific genetic variants illustrates that the prognostic significance of mutations like *TP53* can differ depending on population or ethnic ancestry. A recent study by Seo et al. [22] showed the population-specific influence of germline variants on somatic mutations and outcomes. It was demonstrated that germline variants can influence somatic mutational burden and cancer outcomes. This work revealed that germline variant burden significantly correlated with somatic mutations and worse survival in patients with neuroblastoma [22]. Such findings underscore the importance of exploring and validating somatic mutation effects in diverse populations.

Predictive molecular biomarkers of bladder cancer were described in other genes as well. Mutations in *CDKN2A* were associated with worse recurrence-free survival (HR: 5.76; *p* < 0.001) and worse cancer-specific survival (HR: 2.94; *p* = 0.03) in multivariable analyses [17]. Shao et al. [11] reported that epigenetic-related gene pathway mutations were negatively correlated with tumour recurrence (HR: 0.198; *p* = 0.02), while mutations in *NEB*, *FGFR1*, and *SDHC* were independent predictors of recurrence. Pietzak et al. [10] revealed that *ARID1A* mutations were associated with tumour recurrence in cohorts undergoing BCG therapy. Ward et al. [12] revealed that mutations in *RXRA*, *RHOB*, and *TERT* (promoter) were associated with a significantly decreased time to recurrence, but mutations in *RAS* were associated with better overall survival. In turn, Pang et al.’s [23] study found that *ARID1A* mutations are associated with an increased risk of recurrence after BCG therapy together with high DNA damage repair (DDR) gene alterations in high-risk NMIBC [23]. Our study has not indicated an association between variants in other genes with cancer recurrence. Although research provides different markers of tumour recurrence, further studies are needed on the role of molecular markers in the aetiology of bladder cancer progression.

In breast cancer, mutations in *PIK3CA* also have prognostic relevance and are associated with better invasive disease-free survival (HR: 0.77; *p* < 0.001), with evidence for a more substantial effect in the first 5 years (0 to 5 years: HR: 0.73; *p* < 0.001; 5 to 10 years: HR: 0.82; *p* = 0.037) [24]. However, *PIK3CA* mutations were associated with a lower pathological complete response in HER2-positive breast cancer treated with anti-HER2 therapy. In metastatic breast cancer, *PIK3CA* mutations were associated with worse objective response rate, progression-free survival and time-to-progression [25]. A meta-analysis focused on the prognostic role of *PIK3CA* mutation in colorectal cancer showed that *PIK3CA* mutation has neutral prognostic effects on overall and progression-free survival [26].

Notably, mutations in the *PIK3CA* gene are a molecular marker of targeted therapy and a predictive marker. In vitro studies showed bladder cancer cells with mutations in *PIK3CA* were sensitive to pictilisib compared to wild-type cell lines [27]. In hormone receptor-positive advanced breast cancer women with *PIK3CA* mutations, Alpelisib prolongs progression-free survival [28]. In colorectal cancer, mutations in *PIK3CA* are associated with resistance to therapy by anti-EGFR monoclonal antibodies [29]. However, mutations in PIK3CA may serve as a predictive marker for adjuvant aspirin therapy. In patients with colorectal cancer, mutated-*PIK3CA* was associated with prolonged survival when aspirin was regularly taken [30].

The NGS technique can identify a wide range of variants that could have therapeutic implications. One of the studies identified potentially actionable genetic alternations in 83% of bladder cancer cases that could be useful for treatment decisions for patients with recurrence or metastasis [14]. However, to date, for patients with advanced or metastatic bladder cancer and alterations in the *FGFR2* or *FGFR3* gene, targeted therapy based on the inhibitor of FGFR (erdafitinib) is available [15,31,32]. Bladder cancer is rich in mutations, and new targeted therapies based on NGS results are expected.

A newly developed strategy for identifying genetic alternations based on NGS results in urine samples appears promising for noninvasively detecting bladder cancer. Ward et al. [33] obtained a sensitivity of 87.3% and a specificity of 84.8% for detecting symptomatic bladder cancer using urine samples. Both tumour tissue and urine DNA sample sequencing can be utilised not only for disease diagnosis and treatment decisions but also to assess the response to therapy.

Epigenetic factors such as non-coding RNAs, including long non-coding RNA (lncRNA), microRNA (miRNA) and circular RNA (circRNA), may play a role in bladder carcinogenesis. A study by Zhao et al. [34] demonstrated that the predictive signature based on eight redox-related lncRNAs can independently and accurately predict the prognosis of bladder cancer. As a predicted target gene of MAFG-DT, *IGF2BP2* was highly expressed in tumour tissues, especially in high pathological stages. Spirito et al. [35] researched the involvement of lncRNAs in bladder cancer development as markers for diagnosis and/or follow-up. They suggest that decreased lncRNA Klhl14-AS could promote the early stages of tumour development but not in advanced cancers. Furthermore, they demonstrated reduced expression of another lncRNA gene—RMST in large cancers (>1 cm). They suggest that this lncRNA could play a tumour suppressor role in the late stage, and its loss could affect the progression towards high-grade disease [35].

CircRNAs or miRNAs are also shown to be predictive biomarkers of bladder cancer progression. Shi et al. [36] revealed that circKIF4A sponges miR-375/1231 to promote bladder cancer progression by upregulating *NOTCH2*. MiR-375 was also connected to regulate the expression of *PIK3CA* in the colorectal cancer cell culture model [37]. In NMIBC, an interaction between PIK3CA- and EZH2-signalling pathways was shown. Alterations in the *PIK3CA* gene effect increased the expression of two miRNAs: miR-101 and miR-138, downregulating *EZH2* expression. Moreover, *PIK3CA* alterations facilitate the activation of Akt, which phosphorylates EZH2. Increased expression of miR-101 or miR-138 and the expression of phosphorylated EZH2 are good prognostic factors for the recurrence and progression of NMIBC [38].

Besides genetic or epigenetic factors, lately, researchers have focused on other factors involved in bladder carcinogenesis, such as microbiome and biofilm-associated bacteria. Andolfi et al. [39] proved that increased *Streptococcus* and *Fusobacterium nucleatum* is connected with bladder cancerogenesis. An increased abundance of other bacteria, *Corynebacterium* and *Streptococcus* in men and *Lactobacillus* in women are also seen in patients with bladder cancer [40]. Nardeli et al. have shown an increased abundance of *Porphyromonas* and *Porphyromonas somerae* in bladder cancer patients compared with controls, interestingly related to male patients over 50 years old [41]. Remarkably, the NGS technique allows for searching for molecular- and microbiome-predictive biomarkers for bladder cancer. Integrating molecular and microbiome biomarkers could enhance the accuracy of risk assessment for bladder cancer recurrence.

The main limitation of our study is the number of patients recruited to the study; the number of cases in some subgroups (e.g., subgroups divided according to staging) needed to be larger to perform statistical analyses. More studies in large groups are required to confirm the associations revealed and search for new molecular markers, especially those associated with responses to therapy. Another limitation of our study is that not all possible mutations were revealed in studied genes due to targeted sequencing of hot-spots regions. Whole exome or genomic sequencing could significantly increase the spectrum of mutations.

## 5. Conclusions

Next-generation sequencing enables the search for new molecular markers of bladder cancer progression. Our study revealed that *PIK3CA* mutations are associated with a low risk of bladder cancer recurrence cancer. More research is needed to confirm whether the *PIK3CA* gene is an unquestionable predictive marker of better survival. We also revealed the association of *TP53* mutations with high-grade bladder cancer. Establishing new molecular markers opens the possibility of developing personalised medicine for bladder cancer. Implementing the *PIK3CA* gene as a predictive biomarker in clinical practice requires validation in a large cohort of patients from different populations.

## Figures and Tables

**Table 1:** Characteristics of the patients who participated in the study.

Characteristic	Feature	N (%)
Gender	male	44 (96%)
	female	2 (4%)
Age	mean	69.6 ± SD 10.5
	<60	5 (11%)
	60–70	21 (46%)
	>70	20 (43%)
pT category according to the TNM classification	Ta	5 (10.9%)
	T1	31 (67.4%)
	T2	10 (21.7%)
Histological grade	1	5 (10.9%)
	2	39 (85%)
	3	2 (4.2%)
Recurrence status	no recurrence	24 (52.2%)
	recurrent tumour	22 (47.8%)
	1 recurrence	15 (32.6%)
	2 recurrences	5 (10.9%)
	3 and >recurrences	2 (4.3%)
Cancer-free survival	up to 1 year	15 (32.6%)
	2 years	6 (13%)
	3 years	23 (50%)
	over 3 years	2 (4.3%)
Smoking status	currently	19 (41.3%)
	in the past	18 (39.1%)
	whenever	37 (80.4%)
Exposure to chemicals at work	pesticides	13 (28.3%)
	paints and varnishes	5 (10.8%)
	industrial fumes	3 (6.5%)
	any chemicals	21 (45.6%)
Other cancers		10 (21.7%)

**Table 2:** Variants detected in the bladder tumours.

Gene	Genomic Variant	Protein Alteration	Variant Classification	No. of Cases
*FGFR3*	c.742C>T	p.Arg248Cys	P	1
	c.746C>G	p.Ser249Cys	P	16
	c.1107_1108delGGinsCT	p.Gly370Cys	LP	1
	c.1108G>T	p.Gly370Cys	P	1
	c.1118A>G	p.Tyr373Cys	P	4
	c.1172C>A	p.Ala391Glu	P	1
*PIK3CA*	c.1046G>A	p.Arg349Gln	LP	1
	c.1624G>A	p.Glu542Lys	P	4
	c.1633G>A	p.Glu545Lys	P	6
	c.3140A>G	p.His1047Arg	P	4
*TP53*	c.310C>T	p.Gln104Ter	P	1
	c.517G>A	p.Val173Met	P	2
	c.535C>T	p.His179Tyr	LP	1
	c.637C>T	p.Arg213Ter	P	2
	c.644G>T	p.Ser215Ile	LP	1
	c.722C>T	p.Ser241Phe	LP	1
	c.838A>G	p.Arg280Gly	LP	1
	c.839G>C	p.Arg280Thr	LP	2
*APC*	c.4685A>G	p.Asp1562Gly	VUS	1
	c.4744G>C	p.Ala1582Pro	VUS	2
*FBXW7*	c.1394G>A	p.Arg465His	LP	1
	c.1435C>G	p.Arg479Gly	LP	1
	c.1513C>G	p.Arg505Gly	LP	1
*PTEN*	c.733C>T	p.Gln245Ter	P	1
	c.-1C>G	p.?	VUS	1
*AKT1*	c.49G>A	p.Glu17Lys	VUS	2
*BRAF*	c.1397G>A	p.Gly466Glu	LP	1
	c.1742A>G	p.Asn581Ser	LP	1
*CDKN2A*	c.172C>T	p.Arg58Ter	P	1
	c.323A>C	p.Asp108Ala	VUS	1
*HRAS*	c.37G>C	p.Gly13Arg	P	1
	c.181C>A	p.Gln61Lys	P	1
*KRAS*	c.35G>T	p.Gly12Val	P	2
*NRAS*	c.34G>C	p.Gly12Arg	P	1
	c.182A>G	p.Gln61Arg	P	1
*ATM*	c.8155C>T	p.Arg2719Cys	VUS	1
*CSF1R*	c.890-1G>A	p.?	P	1
*EGFR*	c.1754A>C	p.Tyr585Ser	VUS	1
*ERBB4*	c.2821C>T	p.Gln941Ter	P	1
*FGFR1*	c.541+9G>C	p.?	VUS	1
*KDR*	c.824G>A	p.Arg275Gln	VUS	1
*NOTCH1*	c.7400C>T	p.Ser2467Leu	VUS	1
*PTPN11*	c.1520C>A	p.Thr507Lys	P	1
*RB1*	c.1645C>T	p.His549Tyr	LP	1
*RET*	c.2309G>T	p.Arg770Leu	LP	1
*SMAD4*	c.787+14T>C	p.?	VUS	1
*VHL*	c.416C>G	p.Ser139Cys	VUS	1

P—pathogenic; LP—likely pathogenic; VUS—variants of unknown significance; p.?—an effect on the protein level is expected, but it is not possible to give a reliable prediction of the consequences.

**Table 3:** The spectrum of frequently mutated genes in other research on bladder cancers using next-generation sequencing.

Type of Bladder Cancer	No of Patients	No of the Studied Genes	Genes and Mutation Frequency	Reference
Urothelial Carcinoma in Situ (CIS)	26	31 genes	*TERT* (52%) *TP53* (44%) *ARID1A* (36%) *BRCA2* (28) *KDM* (28%) *ATM* (24%) *BRCA1* (20%)	[5]
Non-Muscle Invasive Tumours	105	341	*TERT* (73%) *FGFR3* (49%) *KDM6A* (38%) *PIK3CA* (26%) *STAG2* (23%) *ARID1A* (21%) *TP53* (21%)	[10]
Non-Muscle Invasive Tumours	58	520	*FGFR3* (48%) *KDM6A* (47%) *KMT2D* (43%) *KMT2C* (34%) *STAG2* (31%)	[11]
Non-Invasive Tumours	956	23	*FGFR3* (95%) *TERT* (77%) *PIK3CA* (56%) *TP53* (41%) *ERCC2* (20%)	[12]
Muscle Invasive Tumours, high-grade	131	18,091	*TP53* (49%) *MLL2* (27%) *ARID1A* (25%) *KDM6A* (24%) *PIK3CA* (20%)	[13]
Advanced urothelial carcinoma (stage IV)	35	182	*PIK3CA* (26%) *CDKN2A*/*B* (23%) *CCND1* (14%) *FGFR1* (14%) *CCND3* (11%) *FGFR3* (11%) *MCL1* (11%) *MDM2* (11%)	[14]
Metastatic urothelial carcinoma	191	591	*TP53* (57%) *KMT2D* (33%) *ARID1A* (29%) *KDM6A* (25%) *RNF213* (20%) *RB1* (18%)	[15]

## Data Availability

The original data presented in the study are openly available in the University of Rzeszow Research Data Repository, http://dx.doi.org/10.15584/ngs.rd.2024, accessed on 8 October 2024.

## Supplementary Materials

The following supporting information can be downloaded at: https://www.mdpi.com/article/10.3390/jcm13247701/s1, Table S1: The verification of variant classification detected in bladder cancer.
